# Unraveling TIMP1: a multifaceted biomarker in colorectal cancer

**DOI:** 10.3389/fgene.2023.1265137

**Published:** 2023-09-29

**Authors:** Xiaode Qiu, Guangqian Quan, Wenquan Ou, Pengfei Wang, Xing Huang, Xinhua Li, Yufan Shen, Weifeng Yang, Jian Wang, Xiaohua Wu

**Affiliations:** ^1^ Department of Clinical Medicine, Fujian Medical University, Fuzhou, China; ^2^ Department of General Surgery, Affiliated Nanping First Hospital, Fujian Medical University, Nanping, China; ^3^ Department of Gastroenterology, Affiliated Nanping First Hospital, Fujian Medical University, Nanping, China; ^4^ Department of Pathology, Affiliated Nanping First Hospital, Fujian Medical University, Nanping, China

**Keywords:** TIMP1, biomarkers, colorectal cancer, immunological microenvironment, drug sensitivity, ferroptosis

## Abstract

**Background:** The pathogenic genes of colorectal cancer (CRC) have not yet been fully elucidated, and there is currently a lack of effective therapeutic targets. This study used bioinformatics methods to explore and experimentally validate the most valuable biomarkers for colorectal cancer and further investigate their potential as targets.

**Methods:** We analyzed differentially expressed genes (DEGs) based on the Gene Expression Omnibus (GEO) dataset and screened out hub genes. ROC curve and univariate Cox analysis of The Cancer Genome Atlas (TCGA) dataset revealed the most diagnostically and prognostically valuable genes. Immunohistochemistry (IHC) experiments were then conducted to validate the expression level of these selected genes in colorectal cancer. Gene set enrichment analysis (GSEA) was performed to evaluate the enriched signaling pathways associated with the gene. Using the CIBERSORT algorithm in R software, we analyzed the immune infiltrating cell abundance in both high and low gene expression groups and examined the gene’s correlation with immune cells and immune checkpoints. Additionally, we performed drug sensitivity analysis utilizing the DepMap database, and explored the correlation between gene expression levels and ferroptosis based on the The Cancer Genome Atlas dataset.

**Results:** The study identified a total of 159 DEGs, including 7 hub genes: SPP1, MMP1, CXCL8, CXCL1, TIMP1, MMP3, and CXCL10. Further analysis revealed TIMP1 as the most valuable diagnostic and prognostic biomarker for colorectal cancer, with IHC experiments verifying its high expression. Additionally, GSEA results showed that the high TIMP1 expression group was involved in many cancer signaling pathways. Analysis of the TCGA database revealed a positive correlation between TIMP1 expression and infiltration of macrophages (M0, M1, M2) and neutrophils, as well as the expression of immune checkpoint genes, including CTLA-4 and HAVCR2. Drug sensitivity analysis, conducted using the DepMap database, revealed that colorectal cancer cell lines exhibiting elevated levels of TIMP1 expression were more responsive to certain drugs, such as CC-90003, Pitavastatin, Atuveciclib, and CT7001, compared to those with low levels of TIMP1. Furthermore, TIMP1 expression was positively correlated with that of ferroptosis-related genes, such as GPX4 and HSPA5.

**Conclusion:** TIMP1 can be used as a biomarker for colorectal cancer and is associated with the immunological microenvironment, drug sensitivity, and ferroptosis inhibition in this disease.

## 1 Introduction

Colorectal cancer (CRC) is a malignant tumor that poses a significant threat to human life and health globally. It has one of the highest incidence and mortality rates among all cancers, ranking third and second, respectively (Sung et al., 2021). Genes play a significant role in the development and progression of CRC (Heide et al., 2022) and are closely linked to cancer pathogenesis, diagnosis, prognosis, the immune microenvironment, and drug responsiveness (Searls, 2003; Ardizzone et al., 2021; Wang et al., 2021). Therefore, correctly identifying effective therapeutic targets for CRC is crucial in enabling early diagnosis and precise management of the condition.

Currently, colonoscopy is the gold standard for diagnosing CRC (Chan and Liang, 2022), but ultimately, its diagnosis still relies on pathological and immunohistochemical results. Therefore, identifying sensitive biomarkers (such as mRNA, miRNA, etc.) in CRC tissue is crucial for the early diagnosis of CRC. Although there are numerous studies exploring biomarkers for CRC (Luo et al., 2021; Malla et al., 2022), many of them have not deeply investigated the clinical significance of genes. Thus, searching for effective biomarkers for CRC patients and exploring their clinical significance remains important and urgent.

In recent years, immunotherapy, drug sensitivity, and ferroptosis analysis have been hotspots in cancer genetics research. Studies have shown that classifying patients according to the types of immune-infiltrating cells and immune-related gene expression may be more helpful for personalized diagnosis and treatment (Bramsen et al., 2017; Pages et al., 2018). Therefore, exploring the correlation between genes and immune-infiltrating cells in CRC patients will provide more appropriate diagnostic and therapeutic guidance. The results of drug sensitivity analysis will provide patients with better drug selection options. Ferroptosis-related analysis provides a basis for studying the pathogenesis of cancer.

**FIGURE 1:**
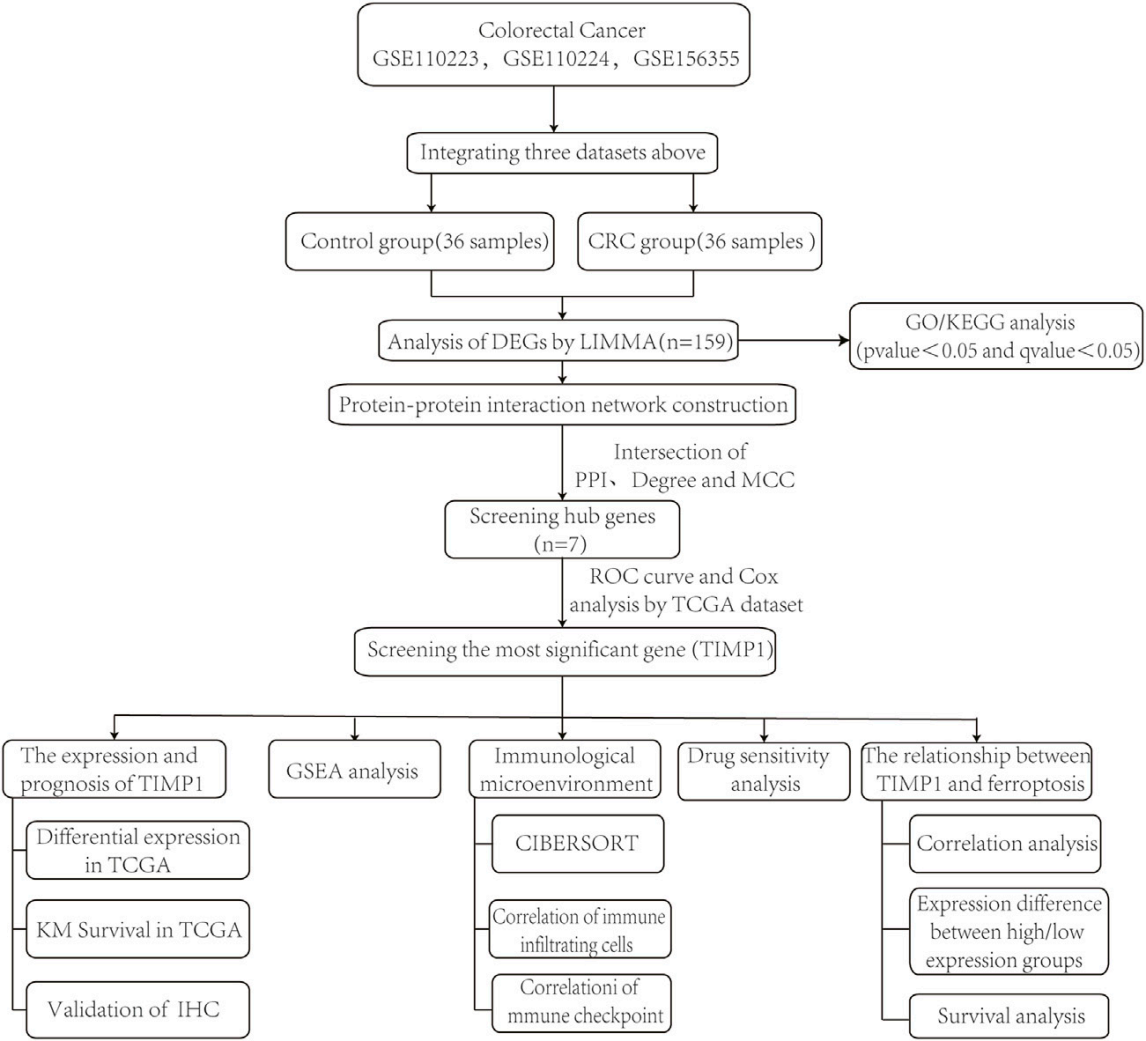
Schematic diagram of the study design.

In this study, we employed bioinformatics techniques to identify differentially expressed genes (DEGs) that were highly expressed in CRC tissues from GEO dataset. We identified 7 hub genes through protein‒protein interaction (PPI) network analysis. Using ROC curve and univariate Cox regression analysis, we identified TIMP1 as the gene with the greatest diagnostic and prognostic value. We subsequently performed survival analysis and conducted gene set enrichment analysis (GSEA) to further investigate the role of TIMP1. We also investigated the relationship between TIMP1 and several factors, including immune infiltration, immune checkpoints, drug sensitivity, and ferroptosis. The study workflow is shown in Figure 1.

## 2 Materials and methods

### 2.1 Obtaining and analysing data

To obtain the gene expression matrix for CRC, we retrieved data from three cohorts (GSE110223, GSE110224, and GSE156355) available on the Gene Expression Omnibus (GEO) database at https://www.ncbi.nlm.nih.gov/geo/. These cohorts included 13, 17, and 6 CRC tissue samples, respectively, along with their corresponding adjacent normal tissues. We combined the data from these three cohorts into a single gene expression matrix using R software (version 4.2.1). For batch correction, we utilized the “ComBat” function from the “sva” package, implementing the Empirical Bayes algorithm. Additionally, we performed data normalization using the “normalizeBetweenArrays” function from the “limma” package, applying the Quantile normalization algorithm. Subsequently, hierarchical clustering was conducted using the “hclust” function from the “stats” package in R, resulting in the generation of a clustering dendrogram. Principal component analysis (PCA) was performed using the “FactoMineR” package, employing the eigendecomposition algorithm, followed by visualization with the “factoextra” package. The PCA plot provided an initial insight into the spatial distribution of cancer tissue and adjacent normal tissue samples.

### 2.2 Screening and analysis of DEGs

We conducted the analysis of differential gene expression using the “limma” package in R. This involved utilizing the “lmFit” function for linear modeling and the “eBayes” function for empirical Bayes statistics. DEGs were identified based on criteria of |log2FC| ≥ 1.5 and adj. *p*-value <0.05. To visually represent our findings, we employed two different packages: “ggplot2” to generate volcano plots and “pheatmap” to create heatmaps. Enrichment analyzes for Gene Ontology (GO) and Kyoto Encyclopedia of Genes and Genomes (KEGG) pathways were carried out on the DEGs using the “clusterProfiler” package. We utilized the “enrichGO” function for GO analysis and the “enrichKEGG” function for KEGG analysis, both employing the hypergeometric distribution test as the statistical method. Significance thresholds were set at *p*-value <0.05 and q value <0.05. Subsequently, we constructed a protein-protein interaction (PPI) network for the DEGs using the STRING online database (https://cn.string-db.org/). Within this network, we identified the top 10 genes with the highest predicted PPI scores. Next, we employed the cytoHubba plugin in Cytoscape software (version 3.9.1) to rank the top 10 genes using the degree and MCC algorithms. Finally, we used the online tool VENNY 2.1, available at https://bioinfogp.cnb.csic.es/tools/venny/index.html, to create a Venn diagram illustrating the intersection of genes identified by the three algorithms. The genes present in this intersection were considered hub genes.

### 2.3 Identification of key genes for CRC diagnosis and prognosis in TCGA dataset

To obtain the necessary data, we accessed the TCGA database (https://cancergenome.nih.gov/) and downloaded gene expression and clinical information. The samples analyzed in this study were derived from the TCGA-COAD and TCGA-READ projects, which included a total of 635 samples. Specifically, there were 584 cancer tissue samples and 51 normal control tissue samples, with 48 of the cancer tissue samples being paired with corresponding adjacent normal tissue samples. To evaluate the diagnostic performance of hub genes in CRC, we used the “pROC” package to generate receiver operating characteristic (ROC) curves for 48 paired cancer and adjacent normal tissues. Using univariate Cox analysis, we screened prognostic genes for CRC in the 584 cancer tissues. We identified TIMP1 as the most valuable gene for diagnosis and prognosis evaluation, and this finding was corroborated through a combination of ROC curve and univariate Cox regression analyzes. We analyzed the difference in TIMP1 expression between the 48 pairs of cancer and adjacent normal tissues using the Wilcoxon rank-sum test. Then, we classified the 584 CRC patients into two groups based on their TIMP1 expression levels, and used Kaplan‒Meier curves to examine the differences in survival rates between them.

### 2.4 Immunohistochemical validation of experimental findings

The CRC tissue microarray (HColA160CS01) used in this study was acquired from Shanghai Outdo Biotech Co., Ltd. It consisted of 80 CRC tissue samples and their corresponding adjacent noncancerous tissues, collected from 43 male and 37 female patients with a mean age of 66 ± 12 years. The ethics committee of the company (Ethics Code: SHYJS-CP-1701008) approved the implementation of the experimental protocols. The use of tissue samples obtained informed consent from the China Human Genetic Resources Administration Office. Our study was conducted in accordance with the Declaration of Helsinki (as revised in 2013). The experimental protocol involved several steps: First, the tissue chips were subjected to baking, dewaxing, and antigen retrieval treatment. Second, we added TIMP1 primary antibody (Proteintech Cat# 16644-1-AP, RRID:AB_2878292, 1:2000) to the chip and incubated it overnight at 4 °C. Afterwards, the chip was exposed to the secondary antibody for 45 min at room temperature, followed by DAB staining and counterstaining with haematoxylin. Finally, we obtained marked images of the slides using an Aperio ScanScope XT Leica (RRID:SCR_018457). We calculated the immunohistochemistry (IHC) total score by multiplying the percentage of positively stained cells with the staining intensity of these cells. We categorized the percentage of positively stained cells into five grades: <5% (0), 5%–25% (1+), 26%–50% (2+), 51%–75% (3+), and 76%–100% (4+). Staining intensity was assessed based on four grades: no staining (0), faint yellow (1+), light brown (2+), and brown (3+). Two experienced pathologists independently interpreted the results. Finally, we analyzed the differences in scores between cancerous and adjacent noncancerous tissue samples by GraphPad Prism software (v 9.3.0).

### 2.5 GSEA

In this section, the TCGA cohort of CRC patients was stratified into two groups based on the median expression level of TIMP1, namely, high and low expression groups. To identify DEGs exhibiting significant variations in expression levels between these two defined groups, the “DESeq2” package was employed. This package utilizes the “DESeq” function, which leverages the negative binomial distribution algorithm to detect genes with statistically significant expression changes under the defined conditions. After identifying the DEGs, we performed a Gene Set Enrichment Analysis using the “GSEA” method from the “clusterProfiler” package in R. The reference gene set used was c2. cp.kegg.v7.5.1. entrez.gmt, with filtering criteria set at ∣NES∣≥1, *p*-value <0.05, and FDR <0.25.

### 2.6 Analysis of the immunological microenvironment

In this section, we conducted an assessment of immune cell infiltration in TCGA cancer tissue samples using the CIBERSORT algorithm, which is based on linear regression. The results of this analysis were visually represented. Subsequently, we employed the “corrplot” package to analyze the relationships among immune cells, utilizing the Pearson correlation coefficient as the statistical measure. To assess differences in immune infiltrating cells between the high and low TIMP1 expression groups within colorectal cancer tissues, we utilized the “ggboxplot” function supplemented by the “wilcox.test” method from the “ggpubr” package. Additionally, we investigated the correlation between TIMP1 expression and immune infiltrating cells using the “cor.test” function from the “stats” package, employing Spearman’s correlation coefficient, and considered statistical significance at a threshold of *p* < 0.05. Furthermore, we used the online platform, Assistant for Clinical Bioinformatics, which is available at https://www.aclbi.com/static/index.html #, to explore the association between TIMP1 and five commonly used immune checkpoint genes (CTLA-4, LAG3, HAVCR2, TNFSF4, and CD160) in TCGA CRC tissues. Finally, we generated box plots illustrating the expression disparities of the five genes between the TIMP1 high and low expression groups within colorectal cancer tissues using the “ggboxplot” function from the “ggpubr” package in R. The statistical analysis supporting these plots involved the application of the Wilcoxon test.

### 2.7 Drug sensitivity analysis

We retrieved drug response data and gene expression data for colorectal cancer cell lines from the DepMap database. Based on the median TIMP1 gene expression values, we categorized the colorectal cancer cell lines into two groups: the TIMP1 high-expression group and the TIMP1 low-expression group. We assessed the sensitivity of the cell lines to drugs using log2FC values, where smaller log2FC values indicate higher sensitivity to the drugs. To determine if there were differences in log2FC values in drug response between the two groups, we performed Mann-Whitney U tests using the “mannwhitneyu” function in Python (v 3.11.3). During the drug screening process, we ensured the simultaneous satisfaction of the following conditions: the median log2FC value for the TIMP1 high-expression group was lower than that of the low-expression group, the median log2FC value for the high-expression group was less than −1, and the Mann-Whitney U test *p*-value between the two cell line groups was less than 0.05.

### 2.8 Ferroptosis correlation analysis

We used the Assistant For Clinical Bioinformatics online platform to analyze the correlation between TIMP1 and five ferroptosis inhibitory genes (GPX4, SLC7A11, HSPA5, HSPB1, and VDAC2) in TCGA CRC tissues. Next, we employed the “ggboxplot” function from the “ggpubr” package in R software to create differential expression boxplots for these five ferroptosis inhibitory genes. These plots allowed us to examine the variation in gene expression levels between the high and low TIMP1 expression groups within colorectal cancer tissues. The statistical analysis underlying these plots was performed using the Wilcoxon test. Finally, we generated Kaplan-Meier curves for these five genes in CRC patients using the GEPIA database (http://gepia.cancer-pku.cn/) to assess their prognostic value.

### 2.9 Statistical methods

In this study, we employed various statistical tools and software for data analysis, including R software (v 4.2.1), Python (v 3.11.3), the STRING database (https://cn.string-db.org/), Cytoscape (v 3.9.1), GraphPad Prism software (v 9.3.0), the Assistant For Clinical Bioinformatics platform (https://www.aclbi.com/static/index.html#/), and the GEPIA database (http://gepia.cancer-pku.cn/).For statistical comparisons between two independent datasets, we applied the Wilcox statistical test. To assess variable correlations, we calculated the Pearson correlation coefficient. Survival and prognosis analysis were conducted using the log-rank test and univariable Cox regression analysis. Statistical significance was defined as *p* < 0.05, and we denoted significance levels as *** for *p* < 0.001, ** for *p* < 0.01, and * for *p* < 0.05.

## 3 Results

### 3.1 GEO dataset integration and analysis

**FIGURE 2:**
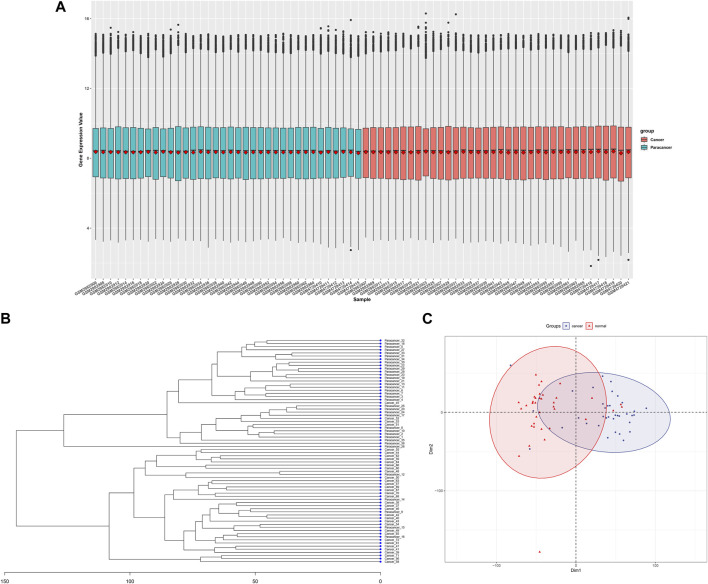
Data processing and initial analysis of merged GSE110223,GSE110224,andGSE156355 datasrts, **(A)** Cancerous and adjacent non-cancerous tissue samples after batch correction and normalization, **(B)** Clustering dendrogram of cancerous and adjacent non-cancerous tissue samples, **(C)** Principal component analysis plot of cancerous and adjacent non-cancerous tissue samples.

After combining three gene expression matrices, a total of 36 cancer tissues and 36 adjacent normal tissues were included. Batch correction and normalization were performed, followed by cluster analysis and principal component analysis. The results showed good intragroup sample correlation, with differences observed between intergroup samples (Figures 2A–C).

### 3.2 Screening DEGs in the GEO dataset

**FIGURE 3:**
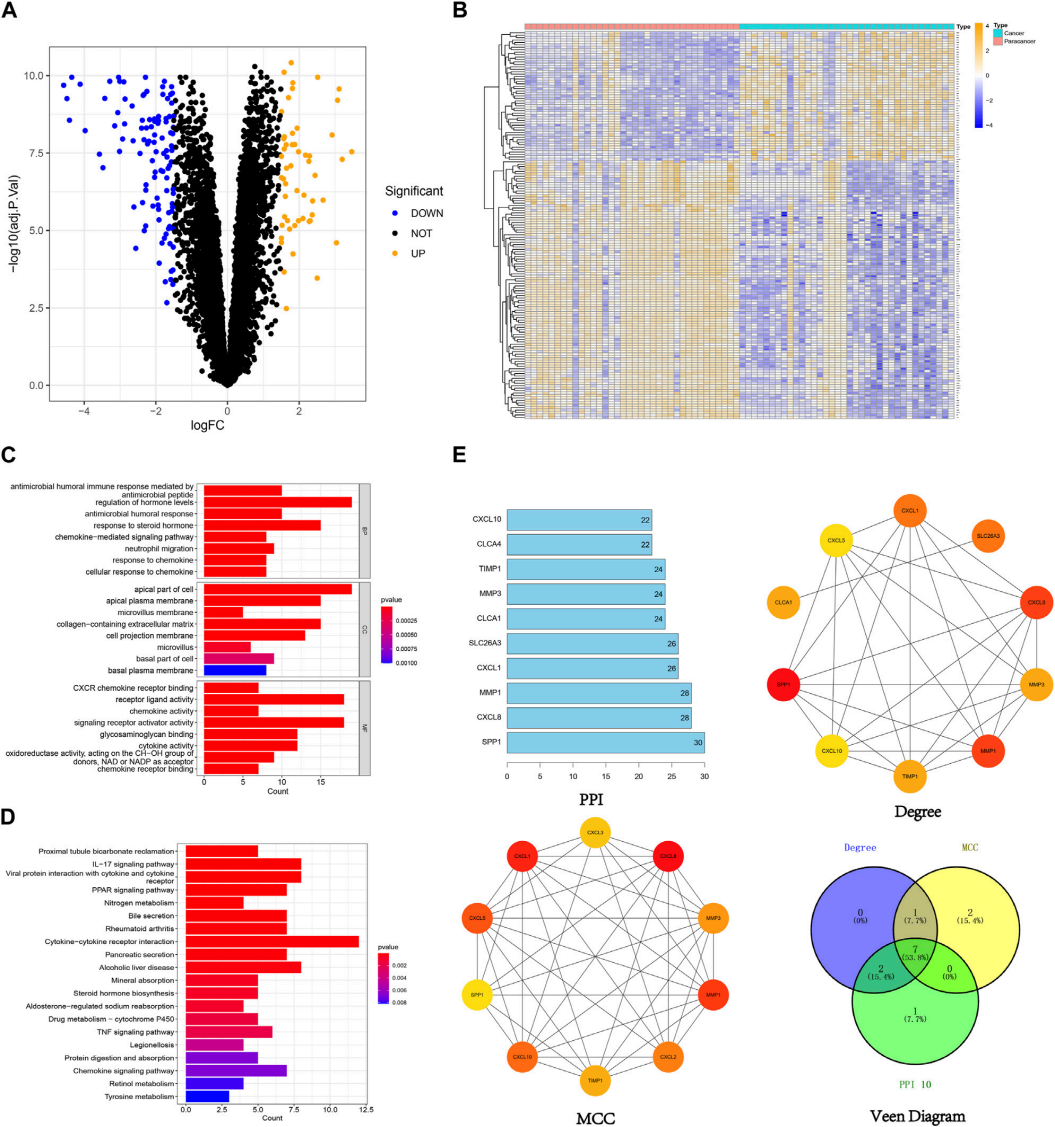
Visualization of 159 DEGs, **(A)** The volcano plot of 159 DEGs (orange dots indicate upregulated genes and blue dots indicate downregulated genes), **(B)** The heatmap of 159 DEGs (orange indicates high gene expression and blue indicates low gene expression), **(C)** GO enrichment analysis of 159 DEGs, **(D)** KEGG enrichment analysis of 159 DEGs, **(E)** Identification of hub genes from 159 DEGs using different algorithms.

In this study, 159 DEGs were screened, including 53 upregulated genes and 106 downregulated genes (Figures 3A, B). Comprehensive details regarding these DEGs can be found in the supplementary document attached to this paper. GO analysis (Figure 3C) unveiled the involvement of DEGs in various vital biological processes, including the regulation of hormone levels, chemokine-mediated signaling pathways, and neutrophil migration. They were also enriched in cellular components such as apical plasma membranes, microvilli and microvillus membranes, extracellular matrix, and basal plasma membranes. Furthermore, the DEGs were found to be associated with molecular functions such as chemokine activity, cytokine activity and receptor ligand activity. The KEGG analysis indicated that the DEGs played crucial roles in essential pathways, such as the chemokine signaling pathway, PPAR signaling pathway, and IL-17 signaling pathway (Figure 3D). After intersecting the results based on the PPI, degree, and MCC algorithms, 7 hub genes were identified: SPP1, MMP1, CXCL8, CXCL1, TIMP1, MMP3, and CXCL10 (Figure 3E).

### 3.3 Diagnostic and prognostic insights of TIMP1 in TCGA CRC analysis

**FIGURE 4:**
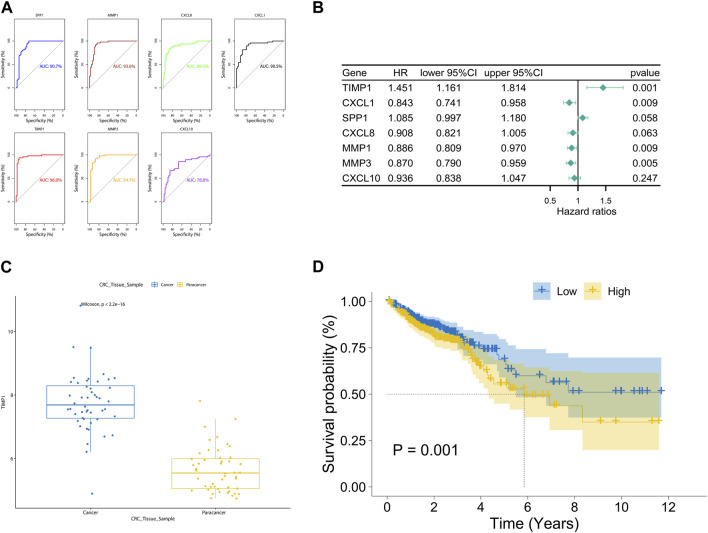
Diagnostic and prognostic value analysis of TIMP1 in TCGA dataset, **(A)** ROC curves and AUC values for seven hub genes, **(B)** Univariate Cox regression analysis of seven hub genes, **(C)** Expression of TIMP1 in cancer and adjacent normal tissue in the TCGA dataset, **(D)** Survival differences between high and low expression groups of TIMP1 in the TCGA dataset.

The area under the ROC curve (AUC) values for SPP1, MMP1, CXCL8, CXCL1, TIMP1, MMP3, and CXCL10 were 0.907, 0.936, 0.895, 0.905, 0.960, 0.947, and 0.788, respectively (Figure 4A). Notably, TIMP1 exhibited the highest AUC value, indicating its significant diagnostic value. By applying a screening threshold of *p* < 0.05 and conducting univariate Cox regression analysis, we identified prognostic genes for CRC, which included TIMP1, CXCL1, MMP1, and MMP3 (Figure 4B). Importantly, TIMP1 showed the strongest correlation with poor prognosis (HR = 1.451, *p* = 0.001). Given TIMP1’s substantial diagnostic and prognostic value, we conducted a comprehensive investigation into its expression. We observed elevated expression of TIMP1 in cancerous tissue samples from the TCGA database compared to adjacent normal tissue samples (Figure 4C). Furthermore, high TIMP1 expression was associated with shorter overall survival time in CRC patients when compared to those with low TIMP1 expression (Figure 4D).

### 3.4 Immunohistochemical validation of TIMP1 expression

**FIGURE 5:**
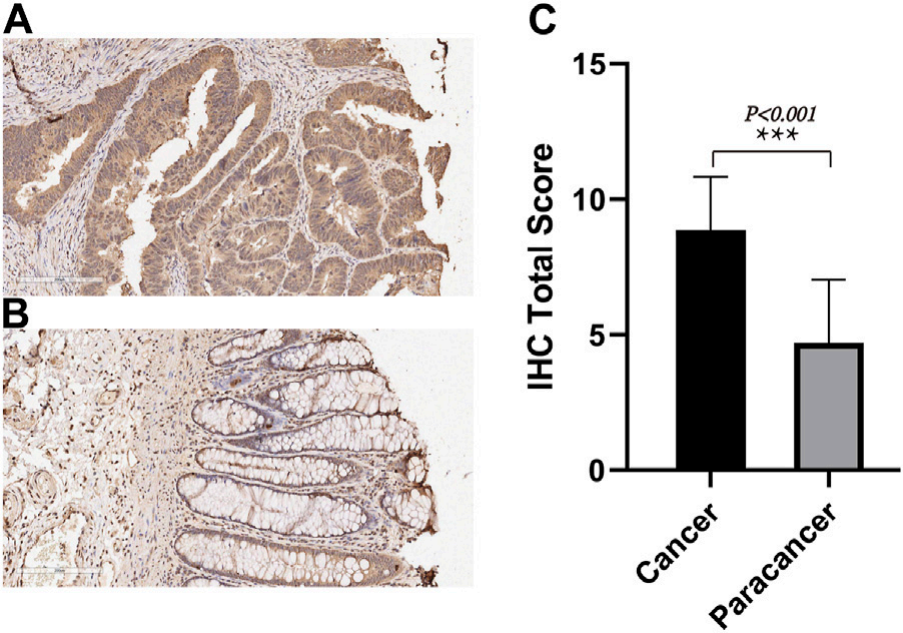
TIMP1 expression in CRC tissues and adjacent normal tissues, **(A)** IHC staining of TIMP1 protein in CRC tissues, **(B)** IHC staining of TIMP1 protein in adjacent normal tissues, **(C)** Semi-quantitative analysis of TIMP1 protein in CRC tissues and adjacent normal tissues by IHC experiments.

We employed IHC methods to assess TIMP1 expression in 80 pairs of CRC tissues and adjacent normal tissues. Ultimately, a total of 71 pairs of tissues were included in the statistical analysis. Our findings revealed a significant upregulation of TIMP1 in CRC tissues compared to their normal counterparts, as visually represented in Figures 5A–C. These results strongly support the potential of TIMP1 as a biomarker for the diagnosis and prognosis of CRC.

### 3.5 GSEA

**FIGURE 6:**
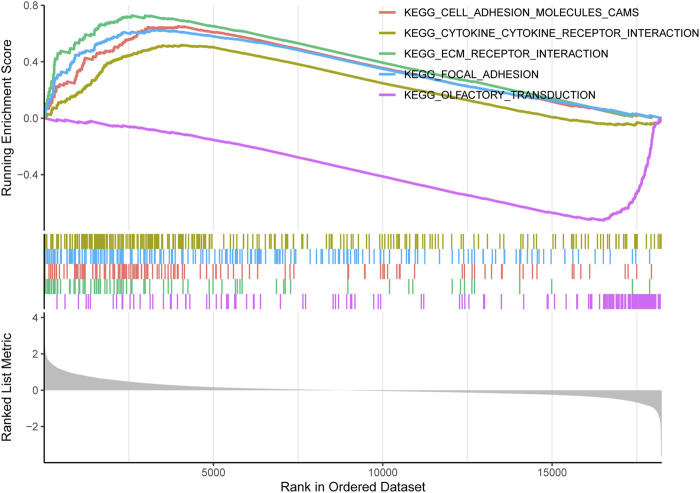
Single-Gene GSEA analysis of TIMP1.

GSEA revealed the pathogenic mechanisms of TIMP1. Our findings indicate that in CRC patients with high TIMP1 expression, the top five enriched pathways identified by pathway analysis were ecm receptor interaction, cell adhesion molecules, focal adhesion, cytokine-cytokine receptor interaction, and olfactory transduction (Figure 6). Notably, olfactory transduction was a downregulated pathway, while the remaining pathways were upregulated.

### 3.6 TIMP1-associated immune landscape in colorectal cancer

**FIGURE 7:**
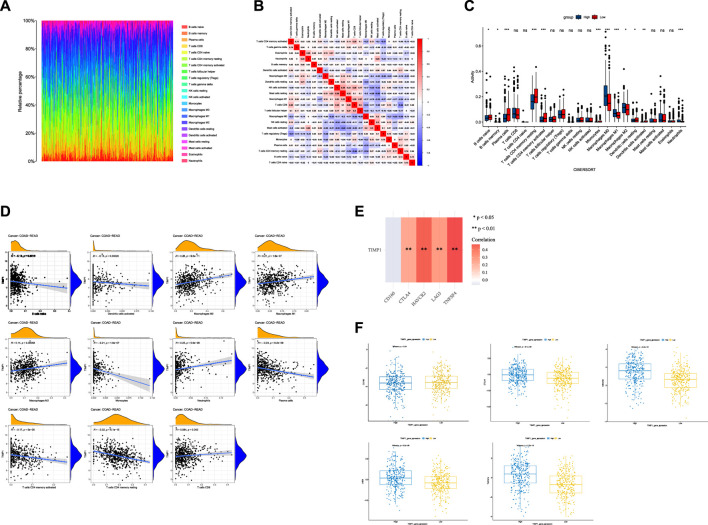
The analysis of the immune microenvironment in CRC tissues from TCGA database, **(A)** Relative proportions of 22 immune cell subtypes in 584 samples analyzed by CIBERSORT algorithm, **(B)** Correlation matrix of immune cell infiltration levels in CRC tissue samples, **(C)** Variations in the 22 subtypes of immune cells in CRC tissues with high and low expression of TIMP1, **(D)** Correlation between TIMP1 expression levels and relative proportions of 11 immune cell types in CRC tissues, **(E)** Association between TIMP1 expression and five commonly expressed immune checkpoint-related genes in CRC using TCGA dataset, **(F)** Differential expression of five immune checkpoint-related genes in CRC tissues with high and low expression of TIMP1.

Using CIBERSORT analysis to assess immune infiltration in CRC tissues from TCGA, we observed variations in the proportions of 22 immune cell types among CRC tissue samples (Figure 7A). In the immune cell correlation matrix (Figure 7B), we noted a significant positive correlation (r = 0.38) between activated NK cells and resting mast cells, while a notable negative correlation (r = −0.44) was found between CD8 T cells and resting memory CD4 T cells. In CRC tissues, higher TIMP1 expression was associated with increased infiltration of M0, M1, and M2 macrophages, as well as neutrophils, but decreased infiltration of monocytes, dendritic cells, memory CD4 T cells, plasma cells, memory B cells, and naive B cells compared to those with lower TIMP1 expression (Figure 7C). Correlation analysis between genes and immune cells indicated a positive association between TIMP1 and the infiltration of M0, M1, and M2 macrophages, neutrophils, as well as CD8 T cells (Figure 7D). Moreover, we observed that TIMP1 expression showed a favorable correlation with the expression of several immune checkpoint genes, including CTLA-4, HAVCR2, LAG3, and TNFSF4 (Figure 7E; Table 1). Differential expression analysis also revealed an increase in the expression of CTLA-4, HAVCR2, LAG3, and TNFSF4 in CRC tissues with high TIMP1 expression (Figure 7F).

**TABLE 1:** Association between TIMP1 expression and immune checkpoint-related genes.

Gene	Checkpoint-releted genes	Cor	P.Value	Pstar
TIMP1	CD160	−0.06	0.11	
TIMP1	CTLA4	0.24	6.92 × 10^−10^	**
TIMP1	HAVCR2	0.45	0	**
TIMP1	LAG3	0.29	4.30 × 10^−13^	**
TIMP1	TNFSF4	0.47	2.08 × 10^−35^	**

### 3.7 Drug sensitivity analysis

**FIGURE 8:**
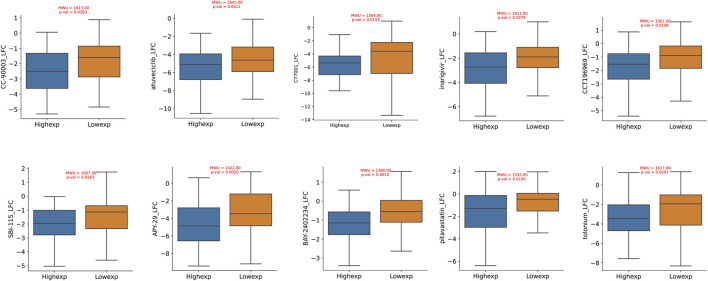
Drug sensitivity differences between CRC cell lines with high and low expression of TIMP1.

Through an exploration of the DepMap database, we conducted a comprehensive investigation into the pharmacological sensitivity of colorectal cancer cell lines, stratified based on the varying expression levels of the TIMP1 gene. Our rigorous inquiry revealed a compelling discovery: colorectal cancer cell lines with elevated TIMP1 expression exhibited significantly increased responsiveness to a selection of ten distinct pharmacological agents (log2FC < −1). In contrast, their counterparts in the TIMP1 downregulated cohort showed comparatively reduced susceptibility to the same spectrum of therapeutic compounds. Importantly, the difference in pharmacological sensitivity between these two distinct clusters demonstrated statistical significance (*p* < 0.05). This curated collection of ten pharmacological agents includes CCT196969, SBI-115, APY-29, BAY-2402234, pitavastatin, tolonium, CC-90003, atuveciclib, CT7001, and inarigivir (Figure 8).

### 3.8 The association between TIMP1 and ferroptosis

**FIGURE 9:**
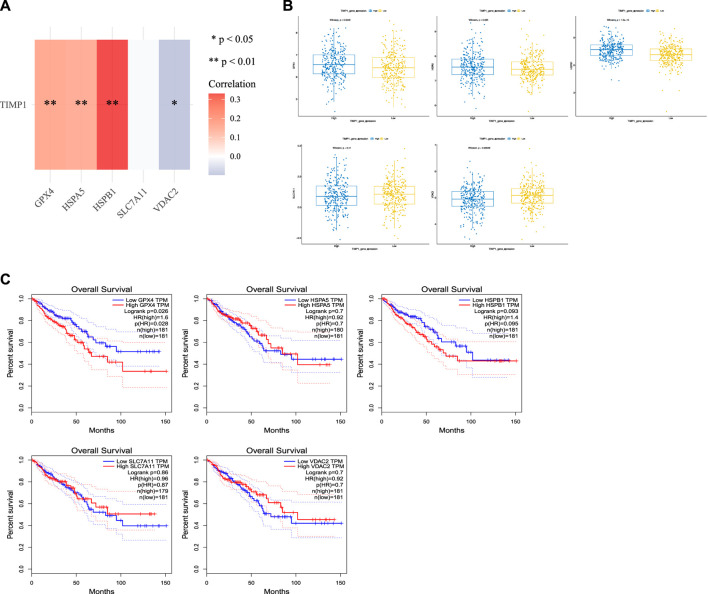
Association between TIMP1 expression and ferroptosis-related genes, **(A)** Association between TIMP1 expression and five commonly expressed ferroptosis-related genes in CRC using TCGA dataset, **(B)** Differential expression of five ferroptosis-related genes in CRC tissues with high and low expression of TIMP1, **(C)** Kaplan-Meier curves for GPX4,HSPA5,HSPB1,SLC7A11, and VDAC2.

We conducted an analysis of the relationship between TIMP1 and common ferroptosis-related genes in CRC tissues from TCGA. Our results demonstrated a positive correlation between TIMP1 expression and the expression of GPX4, HSPA5, and HSPB1 (Figure 9A; Table 2). Differential expression analysis further revealed increased expression of GPX4, HSPA5, and HSPB1 in CRC tissues with high TIMP1 expression, as depicted in Figure 9B. When analyzing the expression levels of genes related to ferroptosis (Figure 9C), it was observed that high GPX4 expression in CRC patients was significantly associated with a poorer survival outcome compared to low expression (*p* = 0.026). Although the survival analysis of HSPB1 did not demonstrate a statistically significant difference (*p* = 0.093), it suggested a trend toward a poorer prognosis. We utilized the online tools Assistant For Clinical Bioinformatics and the GEPIA database to perform the aforementioned analyses.

**TABLE 2:** Association between TIMP1 expression and ferroptosis-related genes.

Gene	Ferroptosis-releted genes	Cor	P.Value	Pstar
TIMP1	GPX4	0.163	4.38 × 10^−5^	**
TIMP1	HSPA5	0.153	1.30 × 10^−4^	**
TIMP1	HSPB1	0.33	3.79 × 10^−17^	**
TIMP1	SLC7A11	−0.008	0.83	*
TIMP1	VDAC2	−0.098	0.1	**

## 4 Discussion

CRC development and progression are influenced by gene expression levels (Chen et al., 2019), suggesting that certain genes may serve as effective diagnostic and therapeutic targets. The pivotal role of TIMP1 in colorectal cancer has been confirmed, as indicated by Beibei Ma et al., who demonstrated its promotion of right-sided CRC cell proliferation and invasion through the activation of the FAK and Akt signaling pathways (Ma et al., 2022). However, for a comprehensive understanding of the unique significance of TIMP1 in colorectal cancer development, systematic bioinformatics analysis is required to delve deeper into its role in the cancer progression. In this study, we conducted differential gene expression analysis on three GEO datasets and confirmed SPP1, MMP1, CXCL8, CXCL1, TIMP1, MMP3, and CXCL10 as core regulatory genes in colorectal cancer through a protein-protein interaction network (Yu et al., 2019). While previous bioinformatics studies typically analyze these regulatory genes as a collective entity, our research adopted a distinct approach, conducting a detailed comparative analysis of these seven genes. ROC curve analysis revealed that TIMP1 exhibited greater precision in distinguishing colorectal cancer tumor tissues from adjacent normal tissues. Additionally, univariate Cox analysis results indicated that TIMP1’s prognostic capability significantly surpassed that of other genes. Consequently, we conclude that TIMP1 holds the highest potential value in the diagnosis and prognosis of colorectal cancer. Our study employed rigorous and systematic screening methods to enhance the credibility of TIMP1’s critical role in colorectal cancer diagnosis and prognosis.

TIMP1 belongs to the family of tissue inhibitors of metalloproteinases (MMPs). Previous studies have demonstrated that extracellular vesicles expressing high levels of TIMP1 can promote distant metastasis in CRC by inducing extracellular matrix (ECM) remodeling (Song et al., 2016; Rao et al., 2022). However, there has been limited research focusing on the other biological functions of TIMP1. In this study, we conducted GSEA of DEGs between CRC tissues with high TIMP1 expression and those with low TIMP1 expression. Our research revealed that TIMP1-associated genes were not only enriched in ECM remodeling but also in cell adhesion molecules (CAMs), focal adhesion, and cytokine-cytokine receptor interactions. While existing studies indirectly suggest that these biological functions are associated with cancer proliferation and metastasis (Araujo et al., 2022; Sowparani et al., 2022; Zhang et al., 2022), they do not specifically address their relationship with TIMP1. Our innovative study deepens the understanding of the functional connections between TIMP1 and the mechanisms underlying cancer pathogenesis.

Recent research has indicated that different types of cancers exhibit distinct patterns of immune cell infiltration, which is closely associated with patients’ chances of receiving immunotherapy (Bai et al., 2021). Furthermore, immune cell infiltration is closely linked to genetics (Liu et al., 2021). Therefore, we employed the CIBERSORT algorithm to investigate the correlation between TIMP1 and immune cell infiltration in CRC patients. Our findings reveal that in CRC tissues with high TIMP1 expression, there is a significant increase in the infiltration of macrophages and neutrophils, suggesting a potentially pivotal role for TIMP1 in immune microenvironment modulation. Based on our discoveries, macrophages can be categorized into M1 and M2 types, where M1 macrophages secrete anti-tumor factors (Jarosz-Biej et al., 2018; Han et al., 2022), while M2 macrophages’ factors may facilitate the progression of colorectal cancer (Zhu et al., 2022). Specifically, in CRC tissues with high TIMP1 expression, there is a higher proportion of M2 macrophage infiltration, implying that TIMP1 may promote the development of colorectal cancer by increasing M2 macrophage infiltration. This study represents the first attempt to stratify immune cell infiltration in CRC patients based on TIMP1 expression levels. Additionally, immune checkpoint blockade is a current focal point in cancer research, as the abnormal activation of immune checkpoint molecules can enable tumor cells to evade immune attacks (Kanani et al., 2021). Prior research did not provide direct evidence of the association between TIMP1 and abnormally activated immune checkpoint molecules. However, our study is the first to confirm a significant positive correlation between TIMP1 expression and various immune checkpoint markers, such as CTLA-4, HAVCR2, LAG3, and TNFSF4. This finding suggests that TIMP1 may play a crucial role in immune regulation.

Considering the significant potential of TIMP1 in the diagnosis and prognosis of colorectal cancer, it is crucial to determine whether it can serve as a viable drug target. However, the current research on TIMP1’s drug potential remains insufficient. Therefore, we utilized the DepMap database to predict which drugs might be more effective in treating colorectal cancer patients with high TIMP1 expression. Our research suggests potential inhibitory effects of CC-90003 and Pitavastatin on colorectal cancer cells. CC-90003, a covalent ERK1/2 inhibitor, disrupts signaling pathways and impacts cell growth and survival, particularly in KRAS-mutant cells (Aronchik et al., 2019), indicating its therapeutic potential in colorectal cancer. Pitavastatin has demonstrated an impact on colorectal cancer behavior by enhancing apoptosis and inhibiting autophagy (Tilija Pun et al., 2022). In addition, Atuveciclib, a selective PTEFb/CDK9 inhibitor, and CT7001, a selective CDK7 inhibitor, are emerging as potential cancer treatment options, as they have entered clinical trials (Lucking et al., 2017; Sava et al., 2020). While their effects on colorectal cancer cells are not-well documented in the current literature, their underlying mechanisms make them promising candidates for therapy. Furthermore, CCT196969, SBI-115, and BAY 2402234 have demonstrated inhibition of melanoma, pancreatic cancer, and myeloid malignancies (Christian et al., 2019; Lei et al., 2022; Reigstad et al., 2022). However, their efficacy against colorectal cancer cells requires further validation due to the diverse behavior of different cancer types. Notably, the roles of Inarigivir, APY-29, and tolonium in cancer therapy remain unexplored in existing literature, necessitating more research to explore their potential applications. There is currently no evidence to suggest a direct association between the mentioned drug mechanisms and TIMP1. However, our innovative research suggests a potential synergistic interaction between TIMP1 and these mechanisms.

Recent cancer research has placed significant focus on ferroptosis, a programmed cell death pathway, which plays a pivotal role in inhibiting tumor growth. Previous studies have indicated that the loss of TIMP1 reduces GPX4 levels, leading to an increase in sorafenib-induced ferroptosis (Wang et al., 2023). Despite these findings, direct evidence linking TIMP1 to other common ferroptosis-related genes has been lacking. Therefore, our study analyzed the expression correlation between TIMP1 and five common ferroptosis-related genes. The research outcomes demonstrate a positive correlation between TIMP1 and GPX4, HSPA5, and HSPB1. GPX4 is a protein associated with poor prognosis, potentially inhibiting ferroptosis in colon cancer cells through specific signaling pathways and interacting synergistically with HSPA5 (Yang et al., 2021; Wang et al., 2022). These findings suggest that the high expression of TIMP1 may interact with GPX4 and HSPA5, making colorectal cancer cells more resistant to ferroptosis. The specific role of HSPB1 in ferroptosis in CRC cells remains unclear, but studies have suggested that the expression of HSPB1 can inhibit ferroptosis in other cancer cells (Sun et al., 2015). While our research has not delved deeply into ferroptosis, these results provide a more comprehensive analysis. Importantly, the association between TIMP1 and ferroptosis-related genes further supports TIMP1 as a potential target for cancer treatment.

In summary, the innovation of our study lies in the rigorous application of bioinformatics analysis methods, which enabled the identification of the TIMP1 gene with the greatest potential within colorectal cancer tissues. We further investigated the relationship between TIMP1 gene expression levels and immune microenvironment, drug sensitivity, and iron death. Our research in these areas demonstrates a high degree of innovation in the study of TIMP1. Nevertheless, we must acknowledge that our study has certain limitations. First, we sourced data for our analysis from public databases, which implies that we were unable to control for the geographic origins and sample types of our data sources. Consequently, the applicability of our research findings may be somewhat limited. Second, in our investigations of immune checkpoints and ferroptosis, we have only provided preliminary indications of a possible association between TIMP1 and these processes. Moreover, further comprehensive investigation is needed to elucidate the precise working mechanisms of TIMP1. Finally, drawing from the existing literature, we constructed the PPI network using the DEGs within this context. We acknowledge that changes in RNA expression do not always directly correlate with protein abundance due to post-transcriptional and translational regulations. However, despite being influenced by these regulatory complexities, these variations can still offer valuable insights into potential biological processes and pathways to a certain extent. Further validation and improvement of these findings will necessitate additional experiments and more comprehensive empirical research.

## 5 Conclusion

In this study, we employed rigorous and scientifically sound methods for data analysis and experimental validation to confirm the significance of TIMP1 in CRC. Furthermore, we explored the association between TIMP1 and key factors such as immune infiltration, immune checkpoints, drug sensitivity, and ferroptosis. Our study reveals that TIMP1 plays a central role in modulating the CRC immune microenvironment by promoting the infiltration of diverse macrophage subpopulations and neutrophils and collaborates with immune checkpoints to regulate patients’ immune responses. Moreover, our results indicate that high TIMP1 expression is associated with sensitivity to ten different drugs and can inhibit CRC cell ferroptosis through the transcriptional activation of genes including GPX4 and HSPA5. Overall, these findings highlight TIMP1 as a valuable diagnostic and prognostic marker for CRC, with a diverse range of biological functions. While TIMP1 may not be a novel biomarker for CRC, our study contributes to a more comprehensive understanding of its biological roles.

## Data Availability

The original contributions presented in the study are included in the article/supplementary material, further inquiries can be directed to the corresponding author.
